# The Effect of Message Framing in Promoting the Mediterranean Diet: The Moderating Role of Eating Self-Efficacy

**DOI:** 10.3390/foods11101454

**Published:** 2022-05-17

**Authors:** Valentina Carfora, Maria Morandi, Patrizia Catellani

**Affiliations:** Department of Psychology, Catholic University of the Sacred Heart, 20123 Milan, Italy; maria.morandi@unicatt.it (M.M.); patrizia.catellani@unicatt.it (P.C.)

**Keywords:** Mediterranean diet, message framing, health messages, prefactual communication, eating self-efficacy

## Abstract

Although a Mediterranean diet (MeDiet) provides several psychophysical health benefits, research on how to effectively promote MeDiet adherence is still lacking. In the present study, we tested the effectiveness of a messaging intervention aimed at promoting the adherence to the Mediterranean diet. A total of 435 Italian participants responded to a questionnaire on their eating self-efficacy and adherence to the MeDiet at Time 1. Then, participants were randomly assigned to three different conditions: (a) gain messages focused on the positive outcomes of MeDiet adherence; (b) non-loss messages focused on the avoided negative outcomes associated with MeDiet adherence; (c) no messages (control). After the 2 week intervention, participants answered some questions regarding their perception of threat and distress, evaluation of the messages, and adherence to the MeDiet at Time 2. We also tested whether the messaging intervention influenced participants’ MeDiet adherence at Time 2. Results confirmed that the messaging intervention enhanced the MeDiet adherence (*F*(2,432) = 4.61; *p* = 0.01, *ηp*^2^ = 0.02), with no difference between exposure to gain or non-loss messages (95% *LLCI* = −0.32; 95% *ULCI* = 0.54). We then tested whether message framing effectiveness was influenced by eating self-efficacy, and results showed that gain messages were more persuasive for participants with low eating self-efficacy (effect size = 0.01; *p* for interaction = 0.03). Discussion suggests that tailoring messages according to receivers’ psychological characteristics seems to be pivotal to enhance the persuasiveness of messages aimed at promoting the MeDiet adherence.

## 1. Introduction

An increasing number of public and social institutions are committed to the promotion of the Mediterranean diet (MeDiet), which received the recognition of UNESCO Intangible Cultural Heritage as it contributes to the protection of the cultural identity of the Mediterranean communities [1]. The eating pattern of the MeDiet suggests eating plant foods (i.e., cereals, fruit, vegetables, pulses, seed, and nuts) and olive oil daily, dairy products (i.e., yoghurt and cheese), fish, seafood, eggs, and poultry a few times per week, and red meat, processed meat, and sweets occasionally [2]. Following these recommendations, people can obtain several physical and mental health benefits, such as a preventive effect against cardiovascular diseases, type 2 diabetes, several types of cancer, cognitive decline, dementia, and depression [3,4]. Moreover, the adherence to the MeDiet is associated with a high perception of physical and mental health, health-related quality of life, and life satisfaction [5,6].

The adherence to the MeDiet is shrinking dramatically. People living in the Mediterranean basin are often not able to follow its recommendations and adopt unhealthy diets, which are rich in animal proteins and precooked food [7]. For instance, more than half of Italians have a low consumption of plant foods and a high consumption of sweets, red meat, and processed meat [8]. Therefore, understanding how to effectively communicate the advantages of the MeDiet adoption is an urgent issue, while also considering the recipients’ capacity to follow the proposed health recommendations. To the best of our knowledge, there are no experimental studies on this topic. With the present study, we aimed to contribute to filling this lacuna by testing the effectiveness of messages about the physical and mental health consequences of the adherence to the MeDiet. We considered the persuasiveness of two types of messages, formulated in terms of either gain (i.e., messages focused on having health benefits) or non-loss (i.e., messages focused on the avoidance of health risks). Moreover, we tested to what extent these messages would be differently persuasive according to receivers’ eating self-efficacy.

## 2. Theoretical Background

### 2.1. Framing Prefactual Messages to Promote Healthy Food Choices

The effectiveness of providing information focused on the psychophysical health consequences of food choices has already been established [9,10,11,12], especially when messages are formulated in prefactual terms (i.e., “if… then” plans) [13,14,15]. The persuasiveness of these messages also varies according to the valence framing of the messages, which may involve the anticipation of positive outcomes associated with the adherence to the recommendation (i.e., positive frame; “if you eat vegetables, you will improve your health”) or the presentation of negative outcomes associated with the non-adherence to the recommendation (i.e., negative frame; “if you do not eat vegetables, you will damage your health”). Positively framed messages appear to be more effective than negatively framed messages in promoting preventive behaviors, including healthy eating [16]. For instance, positive messages are more effective in promoting fruit and vegetable consumption [17,18], as well as sugar-sweetened beverage reduction [19].

Starting from the above, in the present study, we only focused on positively framed messages, leading to the following hypothesis:

**Hypothesis** **1** **(H1).** *Participants who receive positive health messages would have a greater increase in their MeDiet adherence, as compared to participants who receive no messages.*

The framing of the positive valence of a message can be further differentiated by considering the outcome sensitivity level of message framing. Positively framed messages can emphasize either the presence of a positive outcome (i.e., gain message; “if you eat vegetables, you will improve your health”) or the absence of a negative outcome (i.e., non-loss message; “if you eat vegetables, you will avoid harming your health”). On the basis of this classification, past research found that health gain messages are more persuasive than the corresponding non-loss messages [20,21], and the few existing studies on healthy food choices confirmed these results [22,23]. In the present study, we aimed at extending this evidence by comparing the effect of gain and non-loss messages in the case of the adherence to the MeDiet. On the basis of the abovementioned literature, we expected that gain messages would be more effective than non-loss messages. Therefore, we hypothesized the following:

**Hypothesis** **2** **(H2).** *Participants who receive gain health messages would have a greater increase in their MeDiet adherence, as compared to participants receiving non-loss health messages.*

### 2.2. Self-Efficacy

A long tradition of communication research has shown that message persuasiveness is influenced by the individual characteristics of the recipients [24]. Among these, particular attention was paid to self-efficacy, which is defined as the confidence that people have in their ability to perform a certain behavior [25]. Consistently, eating self-efficacy regards individuals’ belief in their ability to successfully control their eating behavior. Having this confidence is of utmost importance because it enhances the likelihood that individuals manage appropriately their diet and translate their intention to eat healthy food into effective actions [26,27]. In the present study, we specifically considered receivers’ eating self-efficacy related to the adherence to the MeDiet [28].

In terms of the effectiveness of health communication, prior research showed that recipients with high self-efficacy are strongly persuaded by health messages, especially when negatively framed [29,30,31]. On the contrary, recipients with low self-efficacy are scarcely persuaded by health messages [29,32,33]. This may be due to the activation of a defense mechanism, which leads them to ignore or reject recommendations asking them to engage in an action they do not feel able to perform. In addition, it is still unclear if people with low self-efficacy are more persuaded by positive or negative message. In this regard, some scholars found no difference in the persuasiveness of these messages [29,32,33], whereas others found that positive messages were more effective than negative messages [31]. However, authors showing the higher effectiveness of positive messages did not consider the difference between gain and non-loss frames. Due to these mixed results, in the present study, we formulated a research question about the moderating effect of self-efficacy in message persuasiveness.

**Research Question 1 (RQ1)**: Which positive frame (i.e., gain or non-loss frame) is more persuasive for people with low eating self-efficacy?

## 3. Materials and Methods

### 3.1. Sample and Procedure

Ethical approval for this study was obtained from the Ethics Committee for Psychology Research of the Catholic University of the Sacred Heart (protocol number: 06–18; approval date: 5 March 2018). We conducted a first power analysis to estimate the sample size needed for the analysis of the variance (ANOVA) to test the effectiveness of the messaging intervention. Considering an effect size f = 0.25, with an alpha = 0.05, power = 0.80, number of groups = 3 (message conditions), number of measurements = 2 (one measure at two timepoints), and *p* = 0.05, the projected sample size needed approximately *n* = 66, with specifically about 22 participants per group. Then, we conducted a second sample size estimation for the moderation analysis aimed at testing the different effects of message framing depending on receivers’ eating self-efficacy. We considered an effect size f = 0.03, as obtained by a previous study on sending WhatsApp messages to increase healthy eating [33]. With an alpha = 0.05, power = 0.80, number of predictors = 3 (condition; eating self-efficacy, condition × eating self-efficacy, and *p* = 0.05, the projected sample size needed for the regressions was approximately *n* = 364, with specifically about 121 participants per group. To ensure this sample size despite any dropouts during the intervention phase and enhance the robustness of our findings, we considered a possible dropout rate of 52%, as obtained in the aforementioned study [33]. Thus, the estimated final sample was 553 participants, with specifically about 184 participants per group.

To collect the data, we asked 80 psychology students to each invite via e-mail or text message three Italian females (one between the age of 18 and 25, one between the age of 26–45, one between the age of 46–75) and three Italian males (same age criteria used for females) to take part in the research. After completing the Time 1 questionnaire, participants were randomly assigned to one of the two message conditions or the control condition (see Section 4.3). To do so, a randomization sequence was created using Microsoft Excel 2016. Participants were allocated to the three conditions in the ratio of 1:1:1. Only participants in the two message conditions received daily messages for 14 days. At the end of the 14 day intervention period, all participants completed the Time 2 questionnaire. Finally, they received feedback on the aims of the study.

**Figure 1:**
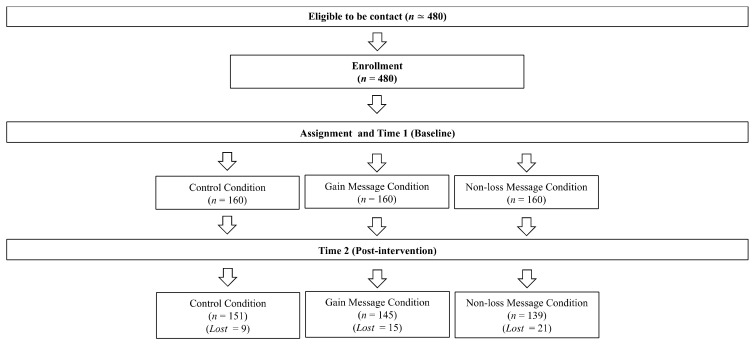
Flow chart of participants’ recruitment.

Figure 1 shows the flow of participants throughout the study. At Time 1, 480 participants fully completed the questionnaire (212 females, 268 males; age range 18–74 years, mean age = 32.49, *SD* age = 13.95). After the messaging intervention, 435 participants correctly filled out the second questionnaire and were retained as the final sample of our study. In sum, we reached 79% of the extended estimated sample size for the moderation analysis (i.e., 435 out of 553 participants); however, this was one and a half times higher than the minimum estimated sample size (i.e., 364 participants).

### 3.2. Measures at Time 1 (T1)

The questionnaire at T1 included several measures. Below, we report the measures relevant to the present paper.

First, we asked participants sociodemographic information including age, gender, level of education, marital status, and demographic dimension of the municipality of residence.

*Adherence to the Mediterranean Diet*. Participants’ past adherence to the MeDiet was assessed using the “Short Questionnaire to Assess Adherence to the Mediterranean Diet” (MEDAS) [34,35,36,37] composed of 14 items (e.g., “How many vegetables serving do you consume per day?”). The final score ranged from 0 to 14. A score smaller than or equal to 5 indicates a low adherence, a score between 6 and 9 indicates a medium adherence, and a score equal to or higher than 10 indicates a high adherence to the MeDiet.

*Eating Self-Efficacy*. Participants’ self-efficacy in the adherence to the MeDiet was measured using an adaptation of the “Self-Efficacy Scale for Adherence to the Mediterranean Diet” (SESAMeD) [28]. The SESAMeD is composed of 22 items rated on a seven-point Likert scale (e.g., “To what extent do you feel confident in your ability to avoid the foods on the following list: Butter, mayonnaise, sugary drinks (soft drinks), etc.; strongly disagree (1)–strongly agree (7)”). High scores correspond to a higher self-efficacy in following the MeDiet (*α* = 0.82).

### 3.3. Messaging Intervention

During the 14 day intervention (between T1 and T2), all participants assigned to the messaging conditions received daily messages via a private chat on WhatsApp. Thus, 14 messages were sent in each condition. All messages were focused on the psychophysical health consequences of the adherence to the MeDiet and were formulated in prefactual terms, presenting a hypothetical future outcome of the recommended behavior (e.g., “if you eat vegetables, you will improve your health”) [13]. Depending on the experimental conditions, messages were formulated either in terms of gain or non-loss. Participants in the *gain message* condition received messages describing the positive psychophysical outcomes of the adherence to the MeDiet (e.g., “if you eat two or three servings of fruit a day, you will feel more energetic” and “if you eat two or three servings of fruit a day, you will strengthen your immune system”). Participants in the *non-loss message* condition received messages focused on the negative psychophysical outcomes avoided by following the MeDiet (e.g., “if you eat two or three servings of fruit a day, you will feel less tired” and “if you eat two or three servings of fruit a day, you will avoid weakening your immune system”). The full list of messages is reported in Appendix A.

### 3.4. Measures at Time 2 (T2)

In the questionnaire at T2, we measured again the adherence to the MeDiet, using the same measures employed at T1. We also included some scales to assess participants’ evaluation of the messages received via WhatsApp.

*Message reading frequency* of all messages received was measured with an item rated on a scale ranging from “never” (1) to “always” (5) (“How many times did you read the messages?”).

*Message tone* was measured with an item using a semantic differential scale ranging from “extremely negative” (1) to “extremely positive” (7) (“How would you rate the tone of the information presented in the messages?”). Higher values indicated a more positive perception of the information tone [37].

*Message involvement* was measured with three items using a Likert scale ranging from “completely disagree” (1) to “completely agree” (7) (e.g., “Messages involved me in what they had to say”; *α* = 0.91) [37].

*Message trust* was measured with three items using a Likert scale ranging from “not at all” (1) to “extremely” (7) (e.g., “How much confidence do you have in the presented information?”; *α* = 0.92) [38].

*Systematic processing* was measured with five items using a Likert scale ranging from “completely disagree” (1) to “completely agree” (7) (e.g., “While I was reading the messages, I thought about what actions I might take based on what I read”; *α* = 0.86) [39].

*Perceived threat to freedom* was measured with four items using a Likert scale ranging from “completely disagree” (1) to “completely agree” (7) (e.g., “The messages I have received have tried to limit my freedom of choice”; *α* = 0.82) [40].

*Identity threat* was measured with four items using a Likert scale ranging from “completely disagree” (1) to “completely agree” (7) (e.g., “The messages I have received have undermined my sense of self-worth”; *α* = 0.72) [41].

*Message-induced distress* was measured with nine items using a Likert scale ranging from “not at all” (1) to “completely” (5) (e.g., “Reading these messages made you feel upset”; *α* = 0.92) [42].

### 3.5. Data Analysis

All analyses were conducted with SPSS 25. We first ran descriptive and correlation analyses to explore the measured variables and the relationships among them. We then checked for the absence of biases in randomization using ANOVA and for biases in dropouts using chi-square test and logistic regression. Next, using independent-sample *t*-tests, we checked if the message reading frequency was influenced by the message frame, and if there were differences in the message evaluation (involvement, trust, systematic processing, identity threat, and message-induced distress) in the two message conditions. As to the main analyses, we used a repeated-measures ANOVA to investigate if the messaging intervention (**H1**) and the message framing (**H2**) were effective in enhancing the adherence to the MeDiet. Finally, to investigate which message frame was more persuasive in increasing the adherence to the MeDiet of participants with low eating-self efficacy (**RQ1**), we ran a moderation analysis using Model 1 of the PROCESS macro for SPSS [43].

### 3.6. Preliminary Analysis

Table 1 reports the sociodemographic characteristics of the final sample. As can be seen in the table, the sample was well balanced in terms of gender. However, most participants were unmarried, young or young adults, with a high school diploma. Table 2 reports the mean and standard deviation of adherence to the MeDiet and eating self-efficacy both in the total sample and among conditions, as well as their correlations.

We then ran two ANOVAs to test if there were differences among conditions on eating self-efficacy and adherence to the MeDiet at Time 1. Results did not show any significant main effect of message conditions on these variables (all *p* > 0.11; all *ηp*^2^ > 0.01). This suggests that randomization was adequate, with the three conditions being comparable on the baseline variables.

Regarding dropouts (Figure 1), 45 participants dropped out at the post-test stage (T2). Chi-square did not show any significant differences in dropouts (*p* = 0.07) across conditions. In addition, results of a logistic regression showed that dropouts did not differ on the basis of eating self-efficacy and adherence to the MeDiet at T1 (all *p* > 0.36). Given the low rate of dropout and the absence of differences in self-efficacy and adherence to the MeDiet at T1, we can assert that the final sample was representative of the initial sample. All the analyses were conducted on the final sample.

## 4. Results

### 4.1. Message Evaluation

First, we verified how frequently participants in the experimental conditions read our messages. Overall, 3.6% of the participants declared they never read or read the messages only few times, 9% declared that they read the messages often, and 87.4% declared that they always read the messages. Using an independent-sample *t*-test, we checked if the message reading frequency was influenced by message frame. Results revealed that gain messages (*M* = 3.89; *SD* = 0.36) were slightly more read than non-loss messages (*M* = 3.78; *SD* = 0.56; *t* = 2.01; *p* = 0.04).

Then, using an independent-sample *t*-test, we checked if there were differences in the message evaluation in the two message conditions. Results showed that participants perceived the tone of non-loss messages (*M* = 5.76; *SD* = 0.91) as more negative compared to the tone of gain messages (*M* = 5.99; *SD* = 0.80; *t* = 2.17, *p* = 0.03). However, there were no differences among conditions (all *p* > 0.31) in participants’ message involvement (*M* = 5.12; *SD* = 1.07), message trust (*M* = 5.21; *SD* = 0.96), systematic processing (*M* = 5.38; *SD* = 0.95), identity threat (*M* = 2.24; *SD* = 0.85), and message-induced distress (*M* = 1.18; *SD* = 0.39). Thus, results showed that all messages were perceived as equally involving, trustworthy, threatening, and stressful, and all of them were equally systematically processed. Table 3 reports the means, standard deviations, and correlations of these message evaluation variables.

### 4.2. Effects of Messages on Adherence to the MeDiet

We then tested whether message intervention changed the adherence to the MeDiet (**H1**) and whether gain messages were more effective than non-loss messages (**H2**).

First, we conducted a 3 (gain message vs. non-loss message vs. control condition) × 2 (T1 vs. T2) repeated-measure ANOVA with adherence to the MeDiet as the dependent variable and repeated measures on the last factor. Results showed a significant effect of condition, *F*(2,432) = 8.25; *p* = 0.001, *ηp*^2^ = 0.04, and a nonsignificant effect of time, *F*(1,432) = 2.58; *p* = 0.11, *ηp*^2^ = 0.01). Moreover, results showed a significant interaction between condition and time*, F*(2,432) = 4.61; *p* = 0.01, *ηp*^2^ = 0.02. Pairwise comparisons showed a significant difference between control (*M* = 5.05; *SD* = 1.62) and both gain (*M* = 6.00; *SD* = 1.86; 95% *LLCI* = −1.09; 95% *ULCI* = −0.25) and non-loss (*M* = 5.75; *SD* = 2.01; 95% *LLCI* = −0.98; 95% *ULCI* = −0.14) message conditions at T2. However, there were no significant differences between gain and non-loss message conditions at T2 (95% *LLCI* = −0.32; 95% *ULCI* = 0.54). We then ran the same analysis excluding participants who did not read the messages often or always. This analysis showed the same results (Appendix B).

In sum, our results supported **H1**, showing that the repeated provision of information on the health consequences of the adherence to the MeDiet was effective in enhancing the adherence to the MeDiet. Moreover, we did not find a significant difference among the two message frames, disconfirming **H2**.

### 4.3. The Moderating Role of Eating Self-Efficacy

**Figure 2:**
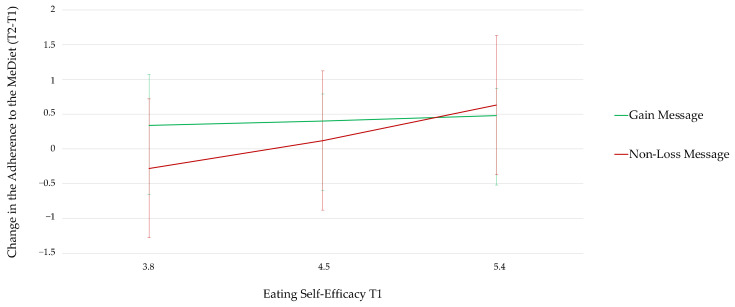
Change in the adherence to the MeDiet according to message framing conditions and participants’ level of eating self-efficacy.

To test which message frame was more effective in increasing the adherence to the MeDiet of participants with low eating-self efficacy (**RQ1**), we calculated the change in the adherence to the MeDiet as a difference score between the adherence at T2 and the adherence at T1. Then, we ran a moderation model with message condition (gain message = 1; non-loss message = 2) as the independent linear variable, eating self-efficacy at T1 as the moderator, and change in the adherence to the MeDiet as the dependent variable (Model 1 of the PROCESS macro for SPSS) [43]. Eating self-efficacy had a significant interaction with message condition (*B* = 0.50; *SE* = 0.24; *t* = 2.08; *95% LLCI* = 0.26; *95% ULCI* = 0.97; Figure 2). Considering participants with low eating self-efficacy, message condition had a significant conditional effect. Thus, participants with low self-efficacy increased their adherence to the MeDiet more in the gain message condition than in the non-loss message condition. Message condition did not have a significant conditional effect in the case of participants with medium or high self-efficacy. Gain and non-loss messages were thus equally effective in enhancing their adherence to the MeDiet (Table 4). An analysis excluding participants who did not read the messages often or always showed the same results (Appendix C).

In sum, regarding **RQ1**, our results showed that gain messages were the most effective messages in enhancing the adherence to the MeDiet of individuals with low eating self-efficacy.

## 5. Discussion

The present study offers two main contributions to research aiming to investigate how to effectively promote the MeDiet. First, testing the effectiveness of a messaging intervention promoting the MeDiet adoption allowed us to extend prior evidence on the effectiveness of positive health messages in promoting single healthy eating behaviors, such as meat reduction [44], consumption of plant-based protein [11], and consumption of fruit and vegetables [17,19]. We showed that positive health messages are also effective in promoting a healthy dietary pattern composed of a variety of eating choices.

Second, testing the effectiveness of our messaging intervention according to receivers’ eating self-efficacy allowed us to show that gain messages are more effective than non-loss messages in the case of individuals with low eating-self efficacy. This is in line with the feature-positive effect, according to which individuals find easier to imagine the presence than the absence of an outcome and, in turn, are more persuaded by messages presenting the presence of an outcome than messages presenting the absence of an outcome [23]. The relevance of this result lies in the fact that low self-efficacy decreases the likelihood that people manage their diet and adopt a healthy diet [26,27]. Therefore, finding messages that are effective in persuading individuals who are not confident in their ability to follow a healthy eating pattern like the MeDiet is of crucial importance.

### 5.1. Limitations and Future Directions

This research had some limitations that future studies might address. Firstly, future research may confirm the effectiveness of positive health messages by measuring their impact on actual behavior, avoiding the possible influence of social desirability and memory biases linked to self-reported behaviors [45]. Secondly, as our sample was composed only of Italian people and was not fully balanced in terms of some sociodemographic variables (e.g., education and age), future research should seek to replicate these findings with other populations. Thirdly, checking whether the effectiveness of this intervention is maintained over time would be very useful. Fourthly, future research could verify whether the effects of gain and non-loss message frames are the same when messages promote outcomes of the adherence to the MeDiet that are not related to health, especially environmental benefits. Lastly, future studies could consider the moderating role of other individual characteristics not considered in the present study, such as health regulatory focus [46], healthy eater self-identity [47], or personal norm [48,49].

### 5.2. Practical Implications

The present study has some practical implications regarding how to devise public communication campaigns and behavioral change interventions. Our findings underlined the potential of instant messaging interventions aimed at promoting healthy diets. In the current study, we found that a relatively simple and low-cost messaging intervention can lead to a significant increase in self-reported adherence to the MeDiet. The limited effect size observed in our study was likely due to the limited duration of our messaging intervention (2 weeks). A substantial shift in participants’ dietary habits usually requires a longer period of time. However, demonstrating for the first time that short messages in prefactual and positive terms can incentivize an initial change in the Italians’ adherence to the MeDiet may be the first step in developing longer promotion campaigns which might support change, while also integrating other types of intervention, such as counseling or coaching.

Lastly, the results of our study open new approaches in the use of public communication campaigns tailored to the receivers’ psychological characteristics. Promotional campaigns might adopt personalized communication using chatbots or smartphone applications which allow measuring receivers’ eating self-efficacy and sending tailored messages on this basis. As shown, both gain and non-loss messages are suitable for individuals with high and medium self-efficacy, while gain messages only are the preferable messages for individuals with low self-efficacy.

## 6. Conclusions

This study supports the effectiveness of repeated provision of positively framed health messages in enhancing the self-reported adherence to the MeDiet. The effectiveness of these messages varied according to receivers’ eating self-efficacy. On the one hand, people with high self-efficacy were convinced to adhere to the Mediterranean diet by a positive frame, regardless of whether it was formulated in terms of gaining positive consequences or avoiding negative consequences. On the other hand, people with low self-efficacy were more persuaded by a gain frame. Thus, tailoring messages according to receivers’ psychological characteristics seems to be pivotal to enhancing the persuasiveness of the messages focused on the promotion of the MeDiet.

## Figures and Tables

**Table 1:** Demographics of study sample.

	Control	Gain Message	Non-Loss Message	Total
**Age**				
% 18–24 years (young)				
Female	54.2	33.8	29.5	40.6
Male	61	30.4	33.3	40.3
% 25–34 years (young adults)				
Female	38.6	23.5	36.1	33
Male	31.2	30.4	27.3	29.5
% 35–54 years (adults)				
Female	4.8	22.1	19.7	14.6
Male	6.5	14.1	22.2	14.9
% 55+ (seniors)				
Female	2.4	20.6	14.8	11.8
Male	1.3	23.9	16.2	14.6
**Number of Residents in Municipality**				
% Less than 5000				
Female	13.3	13.2	8.2	11.8
Male	5.2	23.9	19.2	16.8
% Between 5000 and 10,000				
Female	15.7	19.1	21.3	18.4
Male	26	25	27.3	26.1
% Between 10,000 and 30,000				
Female	19.3	27.9	23	23.1
Male	14.3	20.7	21.2	19
% Between 30,000 and 100,000				
Female	4.8	5.9	11.5	7.1
Male	13	2.2	4	6
% Between 100,000 and 250,000				
Female	1.2	2.9	1.6	1.9
Male	5.2	2.2	3	3.4
% Between 250,000 and 500,000				
Female	32.5	20.6	23	25.9
Male	22.1	20.7	16.2	19.4
% More than 500,000				
Female	13.3	10.3	11.5	11.8
Male	14.3	5.4	9.1	9.3
**Marital Status**				
% Unmarried				
Female	80.7	50	57.4	64.2
Male	77.9	53.3	59.6	62.7
% Married				
Female	1.2	26.5	27.9	17
Male	10.4	33.7	30.3	25.7
% Cohabiting couple				
Female	14.5	14.7	11.5	13.7
Male	11.7	7.6	6.1	8.2
% Separated/Divorced				
Female	3.6	5.9	3.3	3.3
Male	0	5.4	4	3.4
% Widow				
Female	0	1.5	0	1.4
Male	0	0	0	0
**Education**				
% Primary school				
Female	0	0	0	0
Male	0	0	0	0
% Secondary school				
Female	0	1.5	0	0.5
Male	2.6	3.3	2	2.6
% High school, without diploma				
Female	1.2	1.5	1.6	1.4
Male	3.9	8.7	4	5.6
% High school, with diploma				
Female	15.7	25	29.5	22.6
Male	24.7	37	35.4	32.8
% University, without graduation				
Female	38.6	20.6	16.4	26.4
Male	42.9	17.4	14.1	23.5
% University, with graduation				
Female	44.6	51.5	52.5	49.1
Male	26	33.7	44.4	35.4

**Table 2:** Means, standard deviations, and correlations among measured variables in each message condition.

				Control Condition (*n* = 160)		Gain Message Condition (*n* = 160)		Non-Loss Message Condition (*n* = 160)		Total (*n* = 480)	
Study Variables	1.	2.	3.	*M*	*SD*	*M*	*SD*	*M*	*SD*	*M*	*SD*
1. Eating self-efficacy	1			4.64 a	0.75	4.57 a	0.85	4.48 a	0.75	4.57	0.79
2. Adherence to the MeDiet at Time 1	0.38 **	1		5.23 a	1.19	5.56 a	1.82	5.58 a	1.77	5.46	1.63
3. Adherence to the MeDiet at Time 2	0.48 **	0.57 **	1	5.01 a	1.66	5.99 b	1.85	5.75 b	1.99	5.57	1.88

*Note.* M = mean; SD = standard deviation** p < 0.001. The means in a row that do not share the same letter are significantly different from one another.

**Table 3:** Means, standard deviations, and correlations among the message evaluation variables.

Variables	1.	2.	3.	4.	5.	6.	7.	*M*	*SD*
Message involvement	1							5.12	1.07
2. Tone	0.53 **	1						5.87	0.86
3. Trust	0.53 **	0.45 **	1					5.21	0.96
4. Systematic processing	0.63 **	0.36 **	0.42 **	1				5.38	0.95
5. Perceived threat to freedom	−0.16 **	−0.23 **	−0.18 **	−0.16 **	1			2.27	0.99
6. Identity threat	0.05	−0.09	−0.00	−0.00	0.42 **	1		2.24	0.85
7. Message-induced distress	−0.21 **	−0.18 **	−0.16 **	−0.10	0.45 **	0.39 **	1	1.18	0.39

*Note.* ** *p* < 0.001.

**Table 4:** Conditional effects of message condition at different values of the moderator.

	*B*	*SE*	*t*	95% *CI*	
				*LL*	*UL*
**Low eating self-efficacy**					
Message condition	−0.62	0.25	−2.42	−1.14	−0.12
**Medium eating self-efficacy**					
Message condition	−0.28	0.19	−1.45	−0.66	0.10
**High eating self-efficacy**					
Message condition	0.15	0.28	0.55	−0.40	0.71

*Note. B =* regression coefficient; SE = standard error; t = t statistics; CI = confidence interval; LL = lower limit; UL = upper limit.

## Data Availability

The data presented in this study are available on request from the corresponding author.

## Appendix A

Gain Messages	Non-Loss Messages
**Psychological Health Consequences**	
If you eat two or more servings of vegetables per day, you will increase your feeling of well-being.	If you eat two or more servings of vegetables per day, you will reduce your feeling of discomfort.
If you eat two or three servings of fruit per day, you will feel more energetic.	If you eat two or three servings of fruit per day, you will feel less tired.
If you eat two servings of chicken or turkey per week, you will increase your good mood.	If you eat two servings of chicken or turkey per week, you will reduce your bad mood.
If you eat at least three servings of beans, chickpeas, or other legumes per week, you will feel more relaxed.	If you eat at least three servings of beans, chickpeas, or other legumes per week, you will feel less tense.
If you eat three servings of fish or seafood per week, you will increase your concentration.	If you eat three servings of fish or seafood per week, you will reduce your tendency to distraction.
If you eat three or more servings of nuts, almonds, or hazelnuts per week, you will increase your emotional wellbeing.	If you eat three or more servings of walnuts, almonds, or hazelnuts per week, you will reduce your emotional discomfort.
If you drink 2 L of water per day, you will increase your chances of feeling satisfied.	If you drink 2 L of water per day, you will minimize the likelihood of feeling unsatisfied.
**Physical Health Consequences**	
If you eat two or three servings of fruit per day, you will strengthen your immune system.	If you eat two or three servings of fruit per day, you will avoid weakening your immune system.
If you eat two or more servings of vegetables per day, you will offer an adequate number of protective substances to your body.	If you eat two or more servings of vegetables per day, you will avoid offering an inadequate number of protective substances to your body.
If you drink 2 L of water per day, you will help your digestion.	If you drink 2 L of water per day, you will avoid digestion problems.
If you consume olive oil every day for cooking or seasoning, you will live longer.	If you consume olive oil every day for cooking or seasoning, you will avoid shortening your life.
If you eat three or more servings of nuts, almonds, or hazelnuts per week, you will protect your brain function.	If you eat three or more servings of nuts, almonds, or hazelnuts per week, you will avoid damaging your brain function.
If you drink a glass of red wine per day, you will protect your heart.	If you drink a glass of red wine per day, you will avoid damaging your heart.
If you eat at least three servings of beans, chickpeas, or other legumes per week, you will strengthen your muscles.	If you eat at least three servings of beans, chickpeas, or other legumes per week, you will reduce the weakening of your muscles.

## Appendix B

To control the effect of message reading frequency, we conducted a 3 (gain message vs. non-loss message vs. control) × 2 (Time 1 vs. Time 2) repeated-measure ANOVA with adherence to the MeDiet as the dependent variable and repeated measures on the last factor, excluding participants who did not read the messages often or always. Results showed a significant effect of condition*, F*(2,415) = 4.67; *p* = 0.01, *ηp*^2^ = 0.02, and a nonsignificant effect of time, *F*(1,415) = 2.78; *p* = 0.09, *ηp*^2^ = 0.01. Moreover, results showed a significant interaction between condition and time, *F*(2,415) = 4.66; *p* = 0.02, *ηp*^2^ = 0.02. Pairwise comparisons showed a significant difference between control (*M* = 5.05; *SD* = 1.62) and both gain (*M* = 6.04; *SD* = 1.76) and non-loss (*M* = 5.72; *SD* = 2.03) message conditions at T2. However, there was no significant difference between gain and non-loss message conditions at Time 2.

## Appendix C

To control the effect of message reading frequency, we ran a moderation model with message condition (gain message = 1; non-loss message = 2) as the independent linear variable, eating self-efficacy at Time 1 as the moderator, and change in adherence to the MeDiet as the dependent variable (Model 1 of the PROCESS macro for SPSS; Hayes and Preacher, 2013), excluding participants in the message conditions who did not read the messages often or always.

Eating self-efficacy had a significant interaction with messages (*B* = 0.58; *SE* = 0.24; *t* = 2.37; 95% *LLCI* = 0.09; 95% *ULCI* = 1.07). Considering participants with low eating self-efficacy, message condition had a significant conditional effect (*B* = −0.69; *SE* = 0.27, *t* = −2.60; 95% *LLCI* = −0.22; 95% *ULCI* = −0.17). Thus, participants with low self-efficacy increased their adherence to the MeDiet more in the gain message condition than in the non-loss message condition. Considering participants with medium (*B* = −0.28; *SE* = 0.19, *t* = −1.42; 95% *LLCI* = −0.68; 95% *ULCI* = 0.11) and high (*B* = 0.22; *SE* = 0.29, *t* = 0.77; 95% *LLCI* = −0.34; 95% *ULCI* = 0.79) self-efficacy, message condition had no significant conditional effect. Gain and non-loss messages were, thus, equally effective in enhancing the adherence of these participants to the MeDiet.
